# Editorial: 10 years of Frontiers in cell and developmental biology: past discoveries, current challenges and future perspectives

**DOI:** 10.3389/fcell.2025.1625942

**Published:** 2025-06-13

**Authors:** Amanda G. Fisher

**Affiliations:** Department of Biochemistry, Medical Sciences Division, University of Oxford, Oxford, United Kingdom

**Keywords:** chromatin architecture, regulated cell death (RCD), liquid-liquid phase separation (LLPS), embryology, endothelial mechanotransduction

2023 marked the 10th year anniversary of the launch of Frontiers in Cell and Developmental Biology. Our Research Topic celebrates this remarkable milestone by showcasing the work of the journal’s Chief Editors and prominent editorial board members. With an original research paper, a specialty grand challenge, a perspective and four reviews, we highlight some outstanding areas of research that reflect the broad reach of Cell and Developmental Biology.

In their review, Magrath et al. discuss Desmoplastic Small Round Cell Tumor (DSRCT), a rare and highly aggressive pediatric cancer. Given its rarity, for several years model availability was a limiting factor for studying this cancer type but the recent access to new DSRCT samples and cell lines has led to an advance in the field. After explaining the tranlocational origin of a fusion gene between Ewing Sarcoma breakpoint 1 and WT1 DNA-binding domain (EWSR1-WT1) and its oncogenic potential, the authors focus on promising targeted therapeutic strategies. These they divide into two main categories, DSRCT inhibitor targets and immune-based therapy. The authors reflect on the urgent need for more trials of both single and combination therapies, given the new options available and the urgency to advance this field.

A comprehensive review of advances in targeting dysregulated cell death pathways for cancer treatment is provided by Saxena et al. They begin with an overview of how apoptosis can be altered in cancer and outline a series of innovative targeting strategies, such as the use of novel therapeutics like gold complexes, or epigenetic modifying drugs such as 5′ azacytidine. The authors move on to discuss the role of autophagy and summarise an impressive drug panorama of autophagy-targeting drugs that are currently in clinical use, or in clinical/preclinical stages of testing. The review also explores necroptosis and provides details of targeting drugs and their mechanisms of action. The authors helpfully give us insight on the various obstacles that impede the application of necroptosis research for cancer treatment, and stress the necessity for further research in this area. Pyroptosis, a form of regulated cell death that is associated with specific inflammatory responses, is also examined and the exciting potential of combining PD-L1 inhibitors with chemotherapy, or radiation, and using innovative treatments like CAR-T cells to better combat disease, is outlined. New roles of ferroptosis and cuproptosis in cancer research are discussed, and the potential of research in this area to revolutionize cancer treatment is explained. A final section of the review discusses the impact of parthanatos (a cell death mechanism controlled by PARP-1 that is distinct from apoptosis and necroptosis) on carcinogenesis and tumor development. Taken together, this outstanding review highlights the importance that a better understanding the processes of regulated cell death has for future treatment of cancer. It certainly whets the apetite for a deeper understanding of these processes, and illustrates how harnessing combination therapies will improve opportunities to fight cancer.

Liquid-liquid phase separation (LLPS) drives the formation of membraneless intracellular compartments with crucial importance for functional biology. In a review, Xu et al. discuss how aberrant LLPS can influence cancer development and progression, specifically focusing on LLPS and hepatocellular carcinoma (HCC) interplay. To do so, the authors discuss the importance of LLPS in regulating key pathways such as MAPK, cAMP-dependent, and Hippo signaling pathways. They also analyze how LLPS regulates cellular quality control mechanisms including ferroptosis and autophagy. Moreover, they explore the dysregulation of epigenetic factors and the role of RNA in LLPS and HCC. Special attention is given to potential novel treatment strategies for HCC targeting biomolecular condensates and disrupting LLPS. This unique review highlights the involvement of phase separation in HCC and poses the basis for further exploration of drugs that alter phase separation in studies of liver cancer treatment.

Shifting focus to a different field, Kaldis and Porter provide a interesting perspective on ubiquitin ligase research, with a specific focus on Michele Pagano’s many contributions to field. In this perspective, the journey from Pagano’s starting motivation as an endocrinologist in Italy, through his subsequent scientific career is carefully outlined. It is an compelling read, combining personal and scientific opininion, with a particular focus on the stimulating “highs” of collaboration, creating high quality science, publishing, and the thrills of discovery.

In a careful narrative of the embryology field, Brand-Saberi helps us understand the context and significance of her specialty grand challenge, addressing many of the complex ethical considerations that are associated with this field. Brand-Saberi emphasizes the need for international standards and regulations to foster scientific advancement. She mentions the importance of models, while considering the 3R (Replacement, Reduction, Refinment) ethical principles that will lead researchers to develop novel approaches such as organoids and Organ-on-Chip. Although embryology is an evolving field many challenges still persist in understanding the complex signaling pathways, genetic factors, and environmental influences, in addition to technological limitations and increasingly limited teaching in medical and biological education. Without forgetting history, Brand-Saberi reflects on the importance of transparent communication, education and the interdisciplinary nature of this exciting field that holds immense potential.

A detailed review article by Mierke presents the current state of knowledge on the dysregulation of endothelial mechanosensitivity in disease. Several mechanosensors are explored including ion channels, G-Protein-Coupled-Receptors, cell matrix receptors, junctional proteins, and membrane structures. The interplay between endothelial cells and vascular smooth muscle cells is also explored, emphasizing the physiological and pathological consequences of altered mechanotransduction. Future directions for research are highlighted, including advanced models to better mimic the dynamic mechanical environment of blood vessels and deepen our understanding of the fascinating field of endothelial mechanotransduction.

Lastly in this outstanding Research Topic, Ward et al. share their results on how the 3D chromatin architecture of CD4^+^ T cells changes as they transition from activated and memory states. Using a commercial *in situ* Hi-C kit from Arima Genomics, the team successfully generated Hi-C contact maps from as few as 50,000 memory CD4^+^ T cells - a number significantly lower than previously thought necessary. They conducted experiments under three conditions—unstimulated, IL-2 stimulated, and TCR+CD28+IL-2 stimulated—to observe how 3D chromatin in changed and then correlated this to dynamic changes in gene expression. Contrary to the belief that TADs (topologically interacting domains) were static, they found significant changes upon activation with IL-2, which were intensified by additional signaling. This reveals a stepwise refinement of chromatin architecture as mouse CD4^+^ T cells respond to activation, potentially “priming” them for subsequent responses. The paper offers some new insights into T cell activation and its implications for cancer immunotherapy, advancing our understanding of 3D chromatin architecture and T cell functionality.

This Research Topic is certainly a fitting celebration of the journal’s decade of publications, underscoring the breadth and dynamism of the cell and developmental biology fields. It showcases talented scientists and reinforces the importance of their interdisciplinary research. It also illustrates how the journal and the scientists it serves, can work together in concert to support new breakthroughs in understanding and treating complex biological processes. Before closing this editorial it is perhaps worth underscoring some of the challenges facing the core fields of the Journal. These include integrating multi-omics data with dynamic processes captured by increasingly sophisticated imaging, and modelling the *in vivo* complexity of important biological processes in a meaningful and quantitative manner to enable accurate prediction. Alongside this we need to improve our understanding of fundamental cellular and biochemical processes, such as the regulation of intracellular membrane trafficking. There are also clear challenges in standardising widely-used models, such as organoids, induced pluripotent stem cells, and even the diverse impacts of maternal diet and exposures on offspring. Improvements of this sort, that embrace fast moving technical innovations within a coherent conceptual framework, will enable our increasingly interdisciplinary community to thrive over the next decade and beyond. For the Journal to keep pace with these advances, it is critical to recruit accomplished editors and reviewers that can span disciplines, and to continue inspiring emerging researchers–a binary talent that has enriched the Cell and Developmental Biology fields successfully over many years.

